# ﻿Morphology and multi-locus phylogeny reveal four new *Candolleomyces* (Psathyrellaceae) species from Pakistan

**DOI:** 10.3897/mycokeys.121.157282

**Published:** 2025-08-28

**Authors:** Muhammad Haqnawaz, Muhammadah Khalid, Qirui Li, Pingzhu Lu, Aysha Bibi, Arooj Naseer, Najam ul Sehar Afshan, Abdul Rehman Niazi, Abdul Nasir Khalid

**Affiliations:** 1 Chongqing Three Gorges Medical College, Wanzhou Chongqing, 404100, China University of the Punjab Lahore Pakistan; 2 Fungal Biology and Systematics Research Laboratory, Institute of Botany, University of the Punjab, Quaid-e-Azam Campus 54590, Lahore, Pakistan Chongqing Three Gorges Medical College Wanzhou Chongqing China; 3 The High Efficacy Application of Natural Medicinal Resources Engineering Centre of Guizhou Province (The Key Laboratory of Optimal Utilization of Natural Medicine Resources), School of Pharmaceutical Sciences, Guizhou Medical University, Guiyang, Guizhou, 550004, China Guizhou Medical University Guiyang China

**Keywords:** Agaricales, macrofungal diversity, mushrooms, new taxa, Psathyrellaceae

## Abstract

In this study, four new species of the genus *Candolleomyces* are described from Pakistan. Based on morphological and multi-locus phylogenetic analyses, we confirmed the distinction of these taxa. The newly identified species are designated as *Candolleomyces conicus*, *C. denticulatus*, *C. kanhattiensis*, and *C. swaticus*. The distinction of these species from previously known taxa in the genus was verified through comparisons of morphological features and phylogenetic analyses. Three of these species were collected from tropical plains, whereas *C. swaticus* was found in temperate mountainous regions. This distribution highlights the adaptability of the genus to diverse ecological niches across the country. A key to the Pakistani *Candolleomyces* species is also provided.

## ﻿Introduction

The family Psathyrellaceae Vilgalys, Moncalvo & Redhead was established by Redhead et al. (2001), with *Psathyrella* (Fr.) Quél. as the designated type genus. Species within the family have a cosmopolitan distribution, typically growing on decaying logs, humus, or soil, as well as woody debris, in woodlands, lawns, or bogs, and can exhibit either broad or specific substrate relationships (Wächter and Melzer 2020; Wang et al. 2022). The family exhibits remarkable diversity, comprising 20 genera worldwide (Wächter and Melzer 2020; Wang et al. 2022). Among these genera, *Candolleomyces* D. Wächt. & A. Melzer was separated from *Psathyrella* based on multi-locus phylogenetic analyses and the absence of pleurocystidia (Wächter and Melzer 2020). Based on extensive specimen analysis, including morphological and phylogenetic studies, 25 species of *Psathyrella* have been reassigned to *Candolleomyces* (Wächter and Melzer 2020). The genus is characterized by small- to large-sized basidiomata; a veil that is present mainly as fibrillose to scaly or granulose; pleurocystidia absent; clamps present; and a germ pore that is central but often indistinct. Species of this genus are mostly reported from lignicolous, terrestrial, and, rarely, fimicolous habitats. Globally, 73 species have been reported (Ahmad et al. 1997; Ahmad et al. 2024; Han et al. 2024; Cao et al. 2025; Hongsanan et al. 2025; Kiran et al. 2025; Li et al. 2025; Nayana and Pradeep 2025a, b). Asia is the richest continent for *Candolleomyces* diversity, with 51 species, followed by 11 from Europe, six from North America, three from Africa, and one from South America (Haqnawaz et al. 2023b; Ahmad et al. 2024; Han et al. 2024; Kiran et al. 2025; Li et al. 2025; Nayana and Pradeep 2025a, b). In Pakistan, 19 species of the genus have been documented (Ahmad et al. 2024; Han et al. 2024; Haqnawaz et al. 2024a, b; Kiran et al. 2025; Li et al. 2025; Nayana and Pradeep 2025a, b). Taxonomically, this group is challenging due to limitations in phylogenetic analyses, such as inaccurate sequences and small numbers of base pair differences among species, even when multiple regions are used (Yang et al. 2025). This study aims to assess the diversity of macrofungi in general, and specifically of the genus *Candolleomyces*, to facilitate resolution of the genus’s delimitation.

## ﻿Materials and methods

### ﻿Sampling site

Fig. 1

Several basidiomata of *Candolleomyces* were collected from four sites: one in Khyber Pakhtunkhwa (Swat) and three in the Punjab Plains (Khushab, Kot Addu, and Lahore) during the monsoon seasons (July–September) of 2016–2022. The climate of District Swat is moist temperate, with average temperatures ranging from a winter minimum of 4.8 °C to a summer maximum of 33.5 °C and an average annual rainfall of approximately 800 mm. Precipitation occurs mainly in spring and summer, with snowfall at higher elevations. Dominant forest trees include *Abies pindrow* (Royle) Spach, *Pinus wallichiana* A. B. Jackson, and *Diospyros kaki* (Roxb. ex D. Don) G. Don (Sher et al. 2010). Kanhatti Garden, located in Khushab District, experiences hot summers and severe winters, with an overall temperature range of –1 to 50 °C and an average annual rainfall of 103 mm (Shah and Rahim 2017). The climates of Kot Addu and Lahore are very hot during summer and mild in winter, with maximum recorded temperatures of 51 °C and 48.3 °C, respectively, and average annual rainfalls of 127 mm and 575 mm, respectively (Haqnawaz et al. 2023a). The predominant flora of the Punjab Plains consists of *Calotropis procera* (Ait.) Ait. f., *Dalbergia sissoo* Roxb. ex DC., *Diospyros malabarica* (Desr.) Kostel., *Punica granatum* Linn., *Tamarix aphylla* (L.) H. Karst., *Saccharum bengalense* Retz, *S. spontaneum* L., *Syzygium cumini* (L.) Skeels, *Vachellia nilotica* (L.) P.J.H. Hurter & Mabb, and *Zizyphus mauritiana* Lam. (Stewart 1972; Haqnawaz et al. 2023a, b).

**Figure 1.**
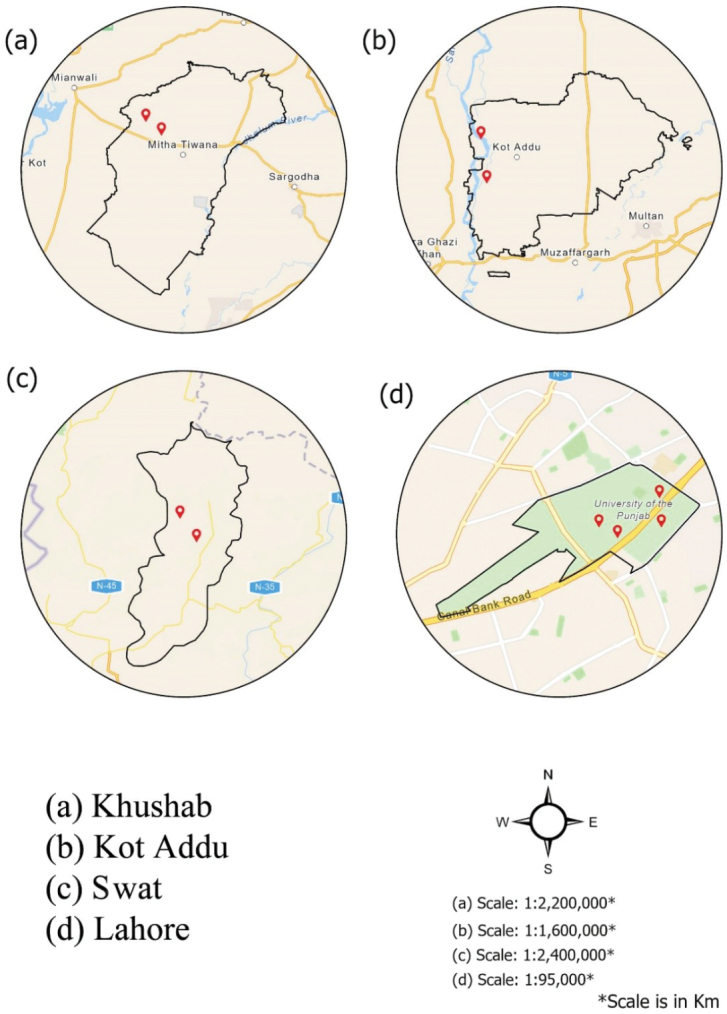
Maps of sampling sites where the novel taxa were found are shown in red.

### ﻿Morphological characterization

Fresh basidiomata were photographed in situ, and information was recorded (Rathnayaka et al. 2025). The specimens were then taken to the laboratory at the Institute of Botany, University of the Punjab, where their morphological characteristics were recorded using the standardized terminology of Vellinga (2001). Color codes were noted according to the Munsell Color Chart (Munsell 1994). All basidiomata were dried either using a hot-air dryer at 30–35 °C or in sunlight, packed in polythene bags, and deposited at the LAH Herbarium, Institute of Botany, University of the Punjab, Quaid–e–Azam Campus, Lahore, Pakistan. Tissues from different parts of the basidiomata were rehydrated in 5% KOH and stained in Congo Red (1% w/v). Microscopic structures were examined using a compound microscope (OLYMPUS BX43, Tokyo, Japan) and measured using ScopeImage 9.0 software, which was connected to the microscope through a digital camera (HDCE–90D). The short form [n/m/p] indicates ‘n’ basidiospores measured from ‘m’ basidiomata of ‘p’ collections. Basidiospore measurements were recorded as (a–) b–c (–d), where a = extreme minimum value; range b–c covers at least 90% of the calculated values; and d = extreme maximum value. ‘L’ denotes length, ‘W’ denotes width, ‘av.L’ denotes average length, and ‘av.W’ denotes average width. ‘Q’ represents the individual spore length/width ratio, while ‘Qav’ represents the average of all Q values (Ge et al. 2010).

### ﻿Molecular phylogenetic analyses

DNA was extracted from the lamellae of dried basidiomata using a modified 2% CTAB protocol (Bruns 1995). The quality of the extracted DNA was evaluated by 1% (w/v) agarose gel electrophoresis (Voytas 2000). The ITS region of the rDNA was amplified using the ITS1F and ITS4 primers; for the LSU region, the LR0R and LR5 primers; and for the *tef*-1α gene, the EF1–983F and EF1–1567R primers were used (Vilgalys and Hester 1990; White et al. 1990; Gardes and Bruns 1993). PCR conditions described by Gardes and Bruns (1993) were carried out with the cycling program as follows: initial denaturation at 94 °C for 2 min; denaturation at 94 °C for 30 s; annealing at 54 °C for 1 min; extension at 71 °C for 2 min; and a final extension at 71 °C for 5 min. Successful amplification of both regions was confirmed by 1% agarose gel electrophoresis using a DNA ladder of 1 Kb and 2 Kb (Usman and Khalid 2020). PCR products for ITS and LSU and DNA products for *tef*-1α rRNA were then sent to the sequencing company Tsing Ke, China.

The final internal transcribed spacer (ITS), large subunit (LSU) ribosomal RNA, and translation elongation factor 1-alpha (*tef*-1α) consensus sequences were obtained by assembling both forward and reverse primers using BioEdit v. 7.2.5 (Hall 1999). The reference sequences used for phylogenetic tree construction were retrieved from the NCBI GenBank database (Sayers et al. 2024). The BLASTn results of close matches showed a similarity of 95% identity or greater, encompassing all published sequences of the genus *Candolleomyces* (Haqnawaz et al. 2024a, b; Li et al. 2025). For the phylogenetic analyses, a Clustal W MUSCLE alignment was implemented in BioEdit v. 7.2.5 with manual adjustments. A combined (ITS–LSU–*tef*-1α) maximum likelihood phylogenetic tree was constructed using RAxML-HPC2 v. 8.2.12 on XSEDE (8.2.10) implemented on the CIPRES Science Gateway (Miller et al. 2010). The GTR+GAMMA nucleotide substitution model was used, with 1,000 bootstrap iterations performed using rapid bootstrapping. Bayesian inference phylogenetic analyses were performed using MrBayes v. 3.2.2 (Ronquist et al. 2012). The model of evolution was estimated by MrModeltest 2.2 (Nylander 2004). Markov chain Monte Carlo (MCMC) sampling in MrBayes v. 3.2.2 (Ronquist et al. 2012) was used to determine posterior probabilities (PP). Six simultaneous Markov chains were run for 1,000,000 generations, and trees were sampled every 1,000^th^ generation. Bootstrap values ≥ 50% and Bayesian PP ≥ 0.90 are indicated on the branches, which were visualized in FigTree v. 1.4.2 (Rambaut 2014). The newly generated sequences were submitted to GenBank, and short descriptions of the species were deposited in MycoBank (Robert et al. 2013).

## ﻿Results

### ﻿Phylogenetic analyses

Fig. 2, Tables 1, 2

**Figure 2.**
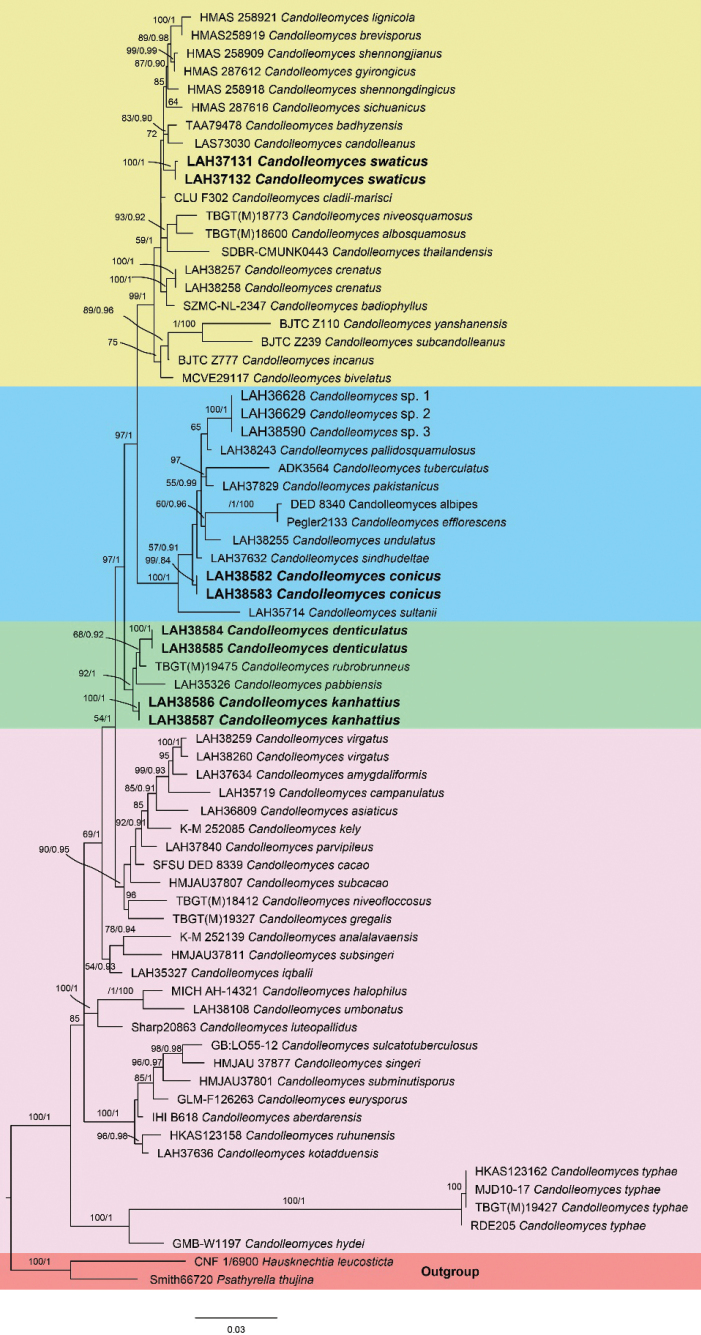
Phylogenetic tree of the genus *Candolleomyces* as generated by maximum likelihood (ML) and Bayesian analyses, based on combined ITS, LSU, and *tef*-1α sequences. Bootstrap values ≥ 50%, based on 1,000 replicates and Bayesian posterior probabilities (PP) ≥ 0.90, are shown at the branches. Novel sequences, generated during this study, are shown in bold.

A combined phylogenetic tree was constructed based on ITS, LSU, and *tef*-1α rRNA sequences, using both maximum likelihood and Bayesian inference methods. All sequences in the dataset had an aligned length of 2,011 nucleotide sites, consisting of 646 from ITS, 864 from LSU, and 501 from *tef*-1α. Among these, 1,441 were conserved sites, 554 were variable, 289 were parsimony-informative, 264 were singletons, and 50 were undetermined characters. The maximum likelihood analysis resulted in a final optimized likelihood value of –8939.483906, and the gamma distribution shape parameter was α = 0.7201. The estimated substitution rate matrix was A–C (1.0625), A–G (1.8756), A–T (1.4431), C–G (0.5381), C–T (4.2531), and G–T (1.0000). The tree comprises 69 sequences of the genus *Candolleomyces*, with two sequences of *Psathyrella thujina* A.H. Sm. and *Hausknechtia leucosticta* (Pat.) Tkalčec, J.Q. Yan, C.F. Nie & C.K. Pradeep as the outgroup. The newly proposed taxa are represented in bold in the final phylogenetic tree (Fig. 2). All four new species are present in three clades: *Candolleomyces conicus* occurs in the blue clade; *C. kanhattiensis* and *C. denticulatus* occur in the green clade; and *C. swaticus* occurs in the yellow clade.

**Table 1.** Sequences used for phylogenetic analyses of *Candolleomyces*, including their current names, locality, voucher specimens, and GenBank accession numbers of the ITS, LSU, and *tef*-1α regions. Sequences generated for this study are shown in bold.

Species Name	Locality	Voucher	ITS	LSU	tef-1α
*Candollemyces aberdarensis*	Kenya	IHI B618	MK421517	MK421517	-
*C. amygdaliformis* T	Pakistan	LAH37634	PP375293	PP375296	-
*C. analalavaensis*	Madagascar	K-M 252139	PQ636530	-	-
*C. albipes*	São Tomé	DED 8340	KX017209	-	-
*C. albosquamosus* T	India	TBGT(M)18600	OQ676549	-	-
*C. asiaticus* T	Pakistan	LAH36809	OK392605	OQ802842	-
*C. badhyzensis* T	Turkmenistan	TAA79478	KC992883	-	-
*C. bivelatus*	Italy	MCVE29117	MF325962	-	MF521811
*C. brevisporus* T	China	HMAS 258919	OR822167	OR822149	OR819986
*C. cacao* T	Sao Tomé	SFSUDED8339	KX017210	-	-
*C. campanulatus* T	Pakistan	LAH35719	OQ308881	OQ802837	-
*C. candolleanus*	Sweden	LAS73030	KM030175	KM030175	-
*C. cladii-marisci* T	Italy	CLU F302	MK080112	-	-
C. conicus T	Pakistan	LAH38582	PV265173	PV265184	PV357393
C. conicus	Pakistan	LAH38583	PV265174	PV265185	PV357394
*C. crenatus* T	Pakistan	LAH38257	PQ329548	PQ329553	PQ369432
*C. crenatus*	Pakistan	LAH38258	PQ329547	PQ329554	PQ369433
C. denticulatus T	Pakistan	LAH38584	PV265175	PV265186	PV357395
C. denticulatus	Pakistan	LAH38585	PV265176	PV265187	PV357396
*C. gregalis*	India	TBGT(M)19327	PQ415499	-	-
*C. gyirongicus* T	China	HMAS 287612	PP734613	PP734624	PP729326
*C. hydei*	China	GMB-W1197	PV434834	PV434836	-
*C. kotadduensis* T	Pakistan	LAH37636	OQ968359	OQ968362	-
C. kanhattiensis T	Pakistan	LAH38586	PV265177	PV265189	PV357397
C. kanhattiensis	Pakistan	LAH38587	PV265178	PV265188	PV357398
*C. kely*	China	K-M 252085	PQ636531	-	-
*C. lignicola* T	China	HMAS 258921	OR822169	OR822151	OR819988
*C. pakistanicus* T	Pakistan	LAH37829	OQ968356	OQ968363	-
*C. efflorescens*	Sri Lanka	Pegler2133	KC992941	-	-
*C. eurysporus* T	Vietnam	GLM-F126263	MT651560	MT651560	-
*C. halophilus* T	Spain	MICH AH-14321	MG825900	-	-
*C. incanus* T	China	BJTC Z777	ON042759	ON042766	ON098509
*C. iqbalii* T	Pakistan	LAH35327	OQ968353	OQ968366	-
*C. luteopallidus* T	USA	Sharp20863	KC992884	KC992884	-
*C. niveofloccosus* T	India	TBGT(M)18412	OQ878345	OR244387	-
*C. niveosquamosus* T	India	TBGT(M)18773	PP741631	PP741635	-
*C. pabbiensis* T	Pakistan	LAH35326	PP058339	PP058345	-
*C. parvipileus* T	Pakistan	LAH37840	OQ968357	OR506278	-
*C. pallidosquamulosus*	Pakistan	LAH38243	PP973161	PP973169	
*C. rubrobrunneus* T	India	TBGT(M)19475	PP741633	PP741636	-
*C. ruhunensis* T	Sri Lanka	HKAS123158	ON685315	-	-
C. swaticus T	Pakistan	LAH37132	PV265182	PV265191	-
C. swaticus	Pakistan	LAH37131	PV265183	PV265190	-
*C. shennongjianus* T	China	HMAS 258909	OR822157	OR822139	OR819976
*C. shennongdingicus* T	China	HMAS 258918	OR822166	OR822148	OR819985
*C. sindhudeltae* T	Pakistan	LAH37632	OQ247908	OQ247912	-
*C. singeri*	China	HMJAU 37877	MW301073	MW301091	MW314080
*C. subcacao* T	China	HMJAU37807	MW301064	MW301092	MW314081
*C. sichuanicus* T	China	HMAS 287616	PP734617	PP734628	PP729330
*C. subcandolleanus* T	China	BJTC Z239	ON042755	ON042762	ON098505
*C. subminutisporus* T	China	HMJAU37801	MW301066	MW301094	MW314083
*C. subsingeri* T	China	HMJAU37811	MG734715	MW301097	MW314085
*C. sulcatotuberculosus*	Germany	GB:LO55-12	KJ138422	-	-
*C. sultanii* T	Pakistan	LAH35714	OQ308835	OQ801565	-
*C. thailandensis* T	Thailand	SDBR-CMUNK0443	MZ146874	-	-
*C. tuberculatus*	Benin	ADK3564	KC992934	-	-
*C. typhae*	China	HKAS123162	ON692696	-	-
*C. typhae*	China	MJD10-17	JX077004	-	-
*C. typhae*	India	TBGT(M)19427	OP225544	-	-
*C. typhae*	China	RDE205	PQ415503	-	-
*C. umbonatus* T	Pakistan	LAH38108	PP058341	PP058347	-
*C. undulatus* T	Pakistan	LAH38255	PQ329546	PQ329551	PQ369428
*C. virgatus* T	Pakistan	LAH38259	PQ329543	PQ329549	PQ369430
*C. virgatus*	Pakistan	LAH38260	PQ329544	PQ329550	PQ369431
*C. yanshanensis* T	China	BJTC Z110	ON042758	ON042765	ON098507
*C.* sp. 1	Pakistan	LAH36628	PV265179	-	-
*C.* sp. 2	Pakistan	LAH36629	PV265180	-	-
*C.* sp. 3	Pakistan	LAH38590	PV265181	-	-
*Psathyrella thujina* (Outgroup)	USA	Smith66720	KC992876	KC992876	-
*Hausknechtia leucosticta* (Outgroup)	India	CNF 1/6900	ON745618	ON745617	ON746005

Notes: Type specimens are marked with T; the symbol “-” indicates no sequence available in GenBank.

**Table 2.** Base pair differences between newly described *Candolleomyces* species and closest sister taxa based on ITS, LSU, and *tef*-1α sequence comparisons.

New Species	Closest Sister Species	Region(s)	Base Pair Differences
*C. conicus*	*C. sindhudeltae*	ITS + LSU	7
	*C. undulatus*	ITS + LSU + *tef*-1α	14
*C. denticulatus*	*C. rubrobrunneus*	ITS + LSU	6
	*C. kanhattiensis*	ITS + LSU + *tef*-1α	10
	*C. pabbiensis*	ITS + LSU	19
*C. kanhattiensis*	*C. rubrobrunneus*	ITS	6
	*C. pabbiensis*	ITS + LSU	13
*C. swaticus*	*C. candolleanus*	ITS	11
	*C. badhyzensis*	ITS + LSU	18

*Candolleomyces conicus* (LAH38582, type) forms a separate branch from its closest species, *C. sindhudeltae* Haqnawaz, Niazi & Khalid (LAH37632) and *C. undulatus* Haqnawaz, Niazi & Khalid (LAH38255), with differences of 7 base pairs (ITS and LSU) and 14 base pairs (ITS, LSU, and *tef*-1α), respectively. *Candolleomyces denticulatus* (LAH38584, type) forms a separate branch from its sister species *C. rubrobrunneus*, *C. kanhattiensis* sp. nov., and *C. pabbiensis*, with differences of 6 base pairs (ITS and LSU), 10 base pairs (ITS, LSU, and *tef*-1α), and 19 base pairs (ITS and LSU), respectively. *Candolleomyces kanhattiensis* (LAH38584, type) forms a distinct branch from its sister species *C. rubrobrunneus* and *C. pabbiensis*, with differences of 6 base pairs (ITS) and 13 base pairs (ITS and LSU), respectively. *Candolleomyces swaticus* (LAH37132) forms a separate branch from its sister species *C. candolleanus* (Fr.) D. Wächt. & A. Melzer (LAS73030) and *C. badhyzensis* (Kalamees) D. Wächt. & A. Melzer (TAA79478), with differences of 11 base pairs (ITS) and 18 base pairs (ITS and LSU), respectively.

### ﻿Taxonomy

#### ﻿ Candolleomyces conicus

Taxon classificationFungiAgaricalesPsathyrellaceae

Haqnawaz, Niazi & Khalid sp. nov.

95D7CEE5-AB59-532D-A7A4-7CDE469569A7

858281

Figs 3, 4

##### Etymology.

The species name “*conicus*” (Latin) refers to the conical pileus.

##### Holotype.

Pakistan • Punjab Province: Lahore District, University of the Punjab, (31°29'39"N, 74°18'06"E, 227 m a.s.l.), on loamy soil, rich in organic matter, under *Dalbergia sissoo*, 10 August 2024, Muhammad Haqnawaz, PU–08 (LAH38582). GenBank: PV265173 [ITS], PV265184 [LSU], PV357393 [*tef*-1α].

##### Diagnosis.

*Candolleomyces conicus* is different from the closest species, *C. sindhudeltae*, by its relatively larger (20–90 mm diam.), conical, plane, reddish gray to light gray pileus with uplifted margins; brownish gray to dull reddish-brown lamellae, a large stipe (50–130 × 4–8 mm), a sheathing annulus with a wavy fibrillous margin, lecythiform, fusiform, tibiform cheilocystidia, and the pileipellis, a transition between hymeniderm and epithelium.

**Figure 3.**
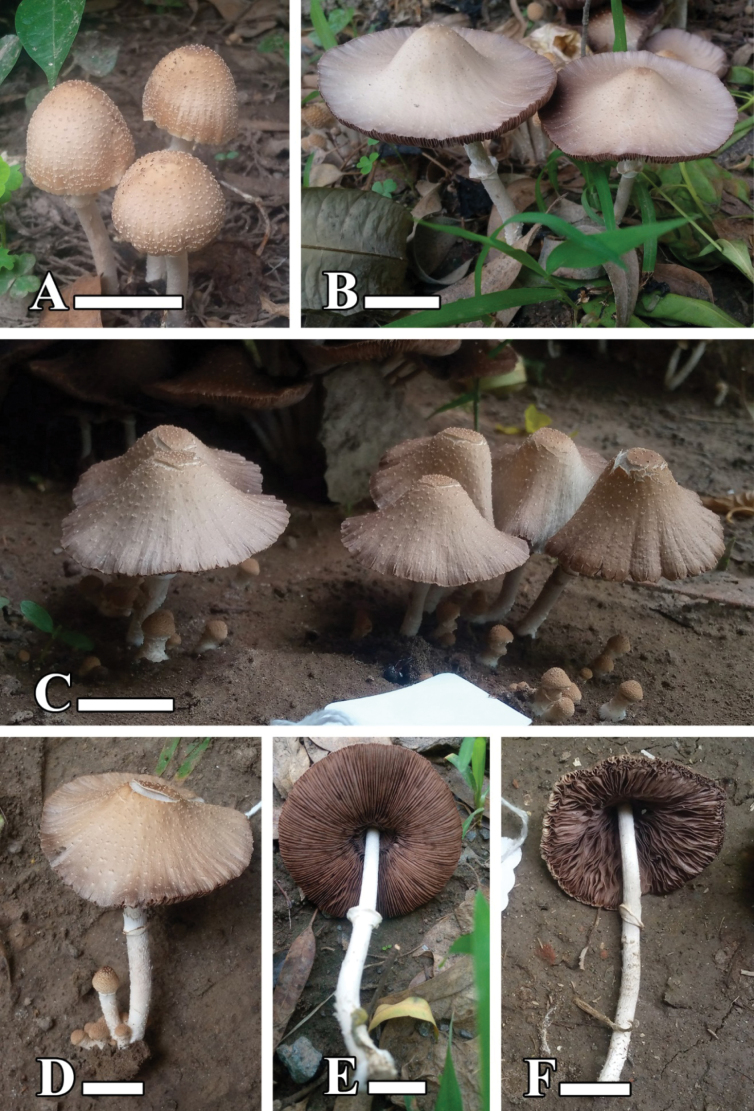
**A–F.** Basidiomata of *Candolleomyces conicus* sp. nov. (holotype LAH38582). Scale bars: 20 mm.

##### Description.

***Pileus*** 20–90 mm diam., parabolic to campanulate with undulating margin in young stages, conical to campanulate than plane with uplifted and undulating margin when old; with a conical umbo, appressed scales, cracked at center, presence of veil elements; dull orange (7.5 YR 7/3) with grayish brown (7.5YR 6/2) center when young, reddish gray (5R 6/1) to light gray (10 YR 8/2) with dull orange (5YR 6/3) center when old, with light gray (5YR 8/1) squamules. ***Lamellae*** adnate, narrow to broad, even, with 3–7 tiers of lamellulae, light brownish (5YR 7/1) when-young, brownish gray (5 YR 6/1) to dull reddish brown (2.5 YR 5/3) when mature. ***Stipe*** 50–130 × 4–8 mm, central, flexuous, tomentose, broad at base and narrow at apex, fibrillose, grayish white (N 8/0) to light gray (7.5 Y 7/2). ***Annulus*** sheathing, wavy and fibrillous at the margin.

**Figure 4.**
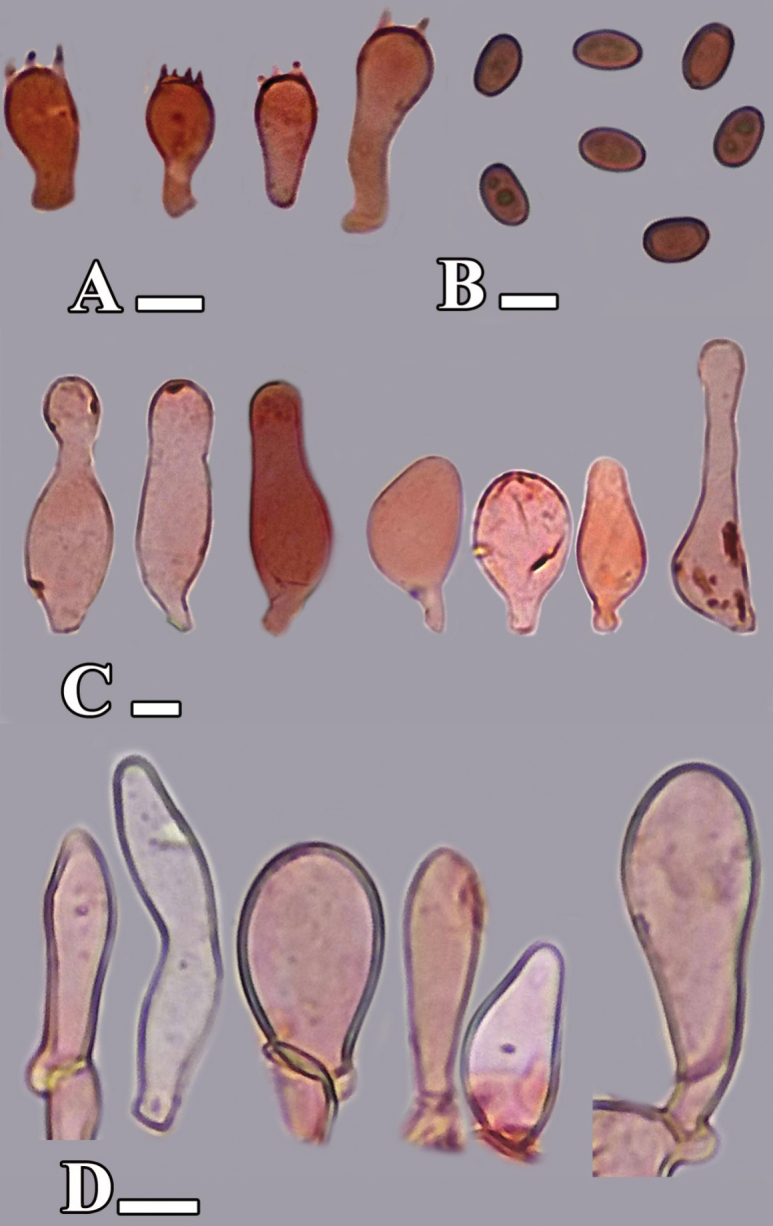
Microscopic structures of *Candolleomyces conicus* sp. nov. (holotype LAH38582). **A.** Basidia; **B.** Basidiospores; **C.** Cheilocystidia; **D.** Caulocystidia. Scale bars: 10 µm (**A–F**).

***Basidiospores*** [200/4/2], (6–)6.5–8.5(–9) × (4–)4.5–5(–6) µm, Q = 1.3–1.8, Qav = 1.65, ellipsoid to amygdaliform, thick–walled, smooth, guttulate, central germ pore present, red in water. ***Basidia*** (12–)13–21(–22) × (6–)7–9(–10) µm, clavate, hyaline in water, thick–walled, smooth, with 2–4 sterigmata. ***Cheilocystidia*** (11–)12–49(–50) × (10–)11–44(–45) µm, utriform, narrowly clavate to broadly clavate, ovoid, globose to subglobose, capitate to lecythiform, cylindrical, oblong, lageniform, flexuose, fusiform, tibiform, hyaline to olive yellow in water, thick-walled. ***Pleurocystidia*** absent. ***Pileipellis*** a transition between hymeniderm and epithelium, one-layered, thin-walled, 36–60 × 29–50 µm, globose to subglobose and ellipsoid to broadly ellipsoid, clavate to broadly clavate, cylindrical cells, hyaline to olive yellow in water. ***Stipitipellis*** a cutis, hyphae subregular, branched 6–17 µm in diameter, thin-walled, septate, and hyaline to yellow in water. ***Caulocystidia*** (18–)19–39(–40) × (9–)10–15(–16) µm, utriform, clavate to broadly clavate, ellipsoid, oblong, hyaline to olive yellow in water. Clamp connections present in all tissues.

##### Ecology and habitat.

Terrestrial, caespitose, on loamy soil rich in organic matter, under the dead trunk of *Dalbergia sissoo*.

##### Additional material examined.

Pakistan • Punjab Province: Kot Addu, Pirhar Gherbi (30°32'48"N, 70°50'40"E, 136 m a.s.l.), on loamy soil, 11 Aug. 2023, Muhammad Haqnawaz, HQ–210 (LAH38583). GenBank: PV265174 [ITS], PV265185 [LSU], PV357394 [*tef*-1α].

##### Notes.

According to phylogenetic analyses, the new species *Candolleomyces conicus* forms a separate clade from its sister species, such as *C. sindhudeltae* and *C. undulatus*. Morpho-anatomically, *Candolleomyces conicus* is different from *C. sindhudeltae* by having a plane, reddish gray pileus with uplifted margins, dull reddish-brown lamellae, a long stipe (50–130 mm length), lecythiform, and tibiform cheilocystidia. *Candolleomyces sindhudeltae* has an umbonate, grayish white, scalloped to cracked pileus with a decurved margin, small stipe (20–35 mm length), ovoid-pedunculate cheilocystidia (Haqnawaz et al. 2023b). *Candolleomyces undulatus* has small (10–27 mm), straight shape, light purplish gray pileus with light brownish gray center, 1–3 tiers of lamellulae, light reddish gray lamellae, small stipe (15–46 × 2–4 mm), cylindrical, spherical with broad peduncle of basidia, ovoid, spheropedunculate, and globose caulocystidia (Li et al. 2025).

#### ﻿ Candolleomyces denticulatus

Taxon classificationFungiAgaricalesPsathyrellaceae

M. Khalid, Haqnawaz & Afshan sp. nov.

A8A3BE21-5D7C-5A36-9EC6-CDEF0D6F6CF7

858282

Figs 5, 6

##### Etymology.

The species name “*denticulatus*” (Latin) refers to the toothed cap margins.

##### Holotype.

Pakistan • Punjab Province: Lahore District, University of the Punjab, (31°29'39"N, 74°18'06"E, 227 m a.s.l.), on loamy soil, rich in organic matter, under *Punica granatum* Linn, 15 Aug. 2024, Muhammadah Khalid, Abdul Nasir Khalid, and Muhammad Haqnawaz, PU–10 (LAH38584). GenBank: PV265175 [ITS], PV265186 [LSU], PV357395 [*tef*-1α].

##### Diagnosis.

*Candolleomyces denticulatus* differs from *C. rubrobruneus* in having toothed cap margins, abundant squamules when young, a grayish-white pileus with a pale orange center, grayish-white edges of the lamellae, and cheilocystidia that are globose, ellipsoid, pedunculate, globose, oblong, or lecythiform; at the same time, caulocystidia are tibiiform, lecythiform, oblong, fusiform, and moniliform.

**Figure 5.**
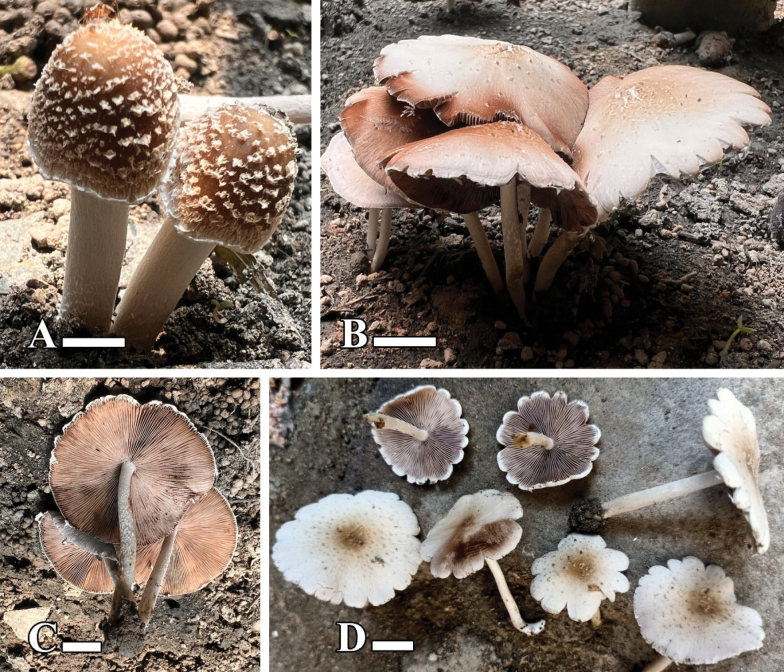
**A–D.** Basidiomata of *Candolleomyces denticulatus* sp. nov. (holotype LAH38584). Scale bars: 10 mm.

##### Description.

***Pileus*** 20–40 mm diam., parabolic in young stages, convex to plane when old, veil remnants present, dense squamules present in young stages, mostly removed when old, margin straight and split in symmetry to toothed, cracked surface, light gray (10 YR 8/1) to grayish white (N 8/0) with dull reddish brown (10 YR 5/4) to pale orange (5 YR 8/3) center, grayish white (N 8/0) squamules. ***Lamellae*** adnate, narrow, even to rarely eroded, with 3–7 tiers of lamellulae, forked, grayish brown (5 YR 6/2) to light brownish gray (5 YR 7/2), edge grayish white (N 8/0). ***Stipe*** 20–30 mm, central, equal, flexuous, surface scabrous, dull reddish brown (10 YR 5/4) to grayish white (N 8/0). ***Annulus*** absent.

**Figure 6.**
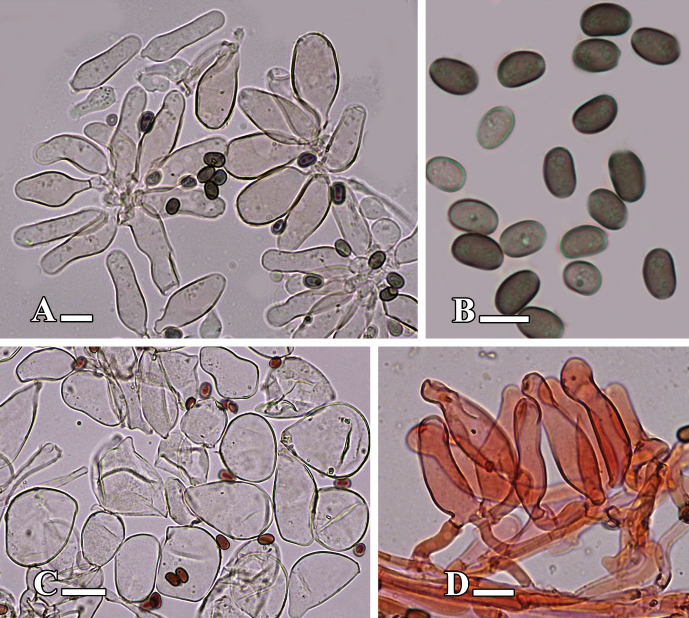
Microscopic structures of *Candolleomyces denticulatus* sp. nov. (holotype LAH38584). **A.** Cheilocystidia; **B.** Basidiospores; **C.** Pileipellis; **D.** Caulocystidia. Scale bars: 10 µm (**A–D**).

***Basidiospores*** [150/3/2], (6.0–)6.5–11.0 (–11.6) × (4.2–)4.5–6.0 (–6.3) µm, Q = 1.2–1.7, Qav = 1.56, amygdaliform in side view, ellipsoid in frontal view, smooth, guttulate, thick-walled, light red in water. ***Basidia*** (11–) 11.9–21.1(–21.5) × (7.5–)7.9–9.1 (–9.2) µm, clavate, hyaline to light gray (5Y 7/1) in 5% KOH, thin-walled, smooth, with 2–4 sterigmata, basal clamp connections present. ***Cheilocystidia*** 25–42 × 8–20 µm, narrowly utriform to broadly utriform, pedunculate, ovoid to conical, globose to subglobose, lecythiform, ellipsoid–pedunculate, narrowly lageniform, thick-walled, hyaline to yellowish gray (2.5Y 5/1) in 5% KOH. ***Pleurocystidia*** absent. ***Pileipellis*** an epitheloid hymeniderm, thick–walled, cells abundantly globose to subglobose, clavate to broadly clavate, ellipsoid to broadly ellipsoid, rarely utriform, oblong, lecythiform, cylindrical, 20–55 × 15–45 µm, hyaline to light gray (5Y 7/1) in 5% KOH. ***Stipitipellis*** made of 4.3–5.4 μm diam., subregular hyphae, rarely branched, hyaline in 5% KOH, thin-walled septate, clamp connections abundant. ***Caulocystidia*** 27–64 × 8–18 µm, utriform to utriform pedunculate, slightly cylindrical, flexuous, ellipsoid, tibiiform, lecythiform, oblong, capitate, fusiform, moniliform, hyaline to light gray (5Y 7/1) in 5% KOH, thick-walled.

##### Ecology and habitat.

Gregarious and caespitose, terrestrial, on alluvial soil rich in organic matter.

##### Additional material examined.

Pakistan • Punjab Province: Kot Addu, Pirhar Gherbi (30°33'07"N, 70°50'40"E, 137 m a.s.l.), on loamy soil, 21 Aug. 2024, Muhammadah Khalid, Abdul Nasir Khalid, and Muhammad Haqnawaz, KA–103 (LAH38585). GenBank: PV265176 [ITS], PV265187 [LSU], PV357396 [*tef*-1α].

##### Notes.

Phylogenetically, *Candolleomyces rubrobrunneus*, *C. pabbiensis*, and *C. kanhattiensis* nov. sp. formed a separate branch from the new taxon *C. denticulatus*. Morpho-anatomically, *Candolleomyces denticulatus* is different from *C. rubrobrunneus* by having a toothed pileus margin, abundant squamules at the immature stage, grayish white pileus with pale orange center, grayish white edges of lamellae, yellowish gray basidiospores, ellipsoid pedunculate, utriform, oblong, lecythiform, cylindrical cheilocystidia; tibiiform, lecytheform, fusiform, moniliform caulocystidia. In contrast, *C. rubrobrunneus* has a straight margin, a dark brown pileus with a brownish, pale, fibrillose to floccose veil, grayish-orange to brown edges of lamellae, olive-brown basidiospores, broadly fusiform capitate cheilocystidia, and subcapitate apex of caulocystidia (Nayana and Pradeep 2024). However, *C. pabbiensis* is distinguished from our new taxon by conical to broadly parabolic pileus with straight margins, grayish white lamellae, light brown basidiospores, and plump cheilocystidia with an obtuse to subobtuse apex (Haqnawaz et al. 2024b). *Candolleomyces kanhattiensis* differs from the new taxon *C. denticulatus* by having a sub-globose to campanulate pileus with decurved, undulating margins, a shiny stipe with a clavate base, greenish gray basidiospores, and cylindrical, ellipsoid, and globose cheilocystidia (in this study).

#### ﻿ Candolleomyces kanhattiensis

Taxon classificationFungiAgaricalesPsathyrellaceae

Bibi, Haqnawaz, Afshan & Khalid sp. nov.

4A084D8F-3EA7-52A0-9C28-0DE6BC3CECEC

858285

Figs 7, 8

##### Etymology.

The species name “*kanhattiensis*” (Latin) refers to the type locality of the species, ‘Kanhatti Garden’, Khushab, Pakistan.

##### Holotype.

Pakistan • Punjab Province: Khushab District, Kanhatti Garden (32°25'22"N, 72°14'59"E, 555 m a.s.l.), on loamy soil, rich in organic matter, under *Phoenix dactylifera*, 15 Aug. 2022, Ayesha Bibi, KUN–03 (LAH38586). GenBank: PV265177 [ITS], PV265189 [LSU], PV357397 [*tef*-1α].

##### Diagnosis.

This species is different from its closest species, *Candolleomyces rubrobrunneus*, by having sub-globose to campanulate, dull orange pileus with decurved margins, shiny stipe with clavate base, light brownish gray basidiospores, and cylindrical, ellipsoid, and globose cheilocystidia.

**Figure 7.**
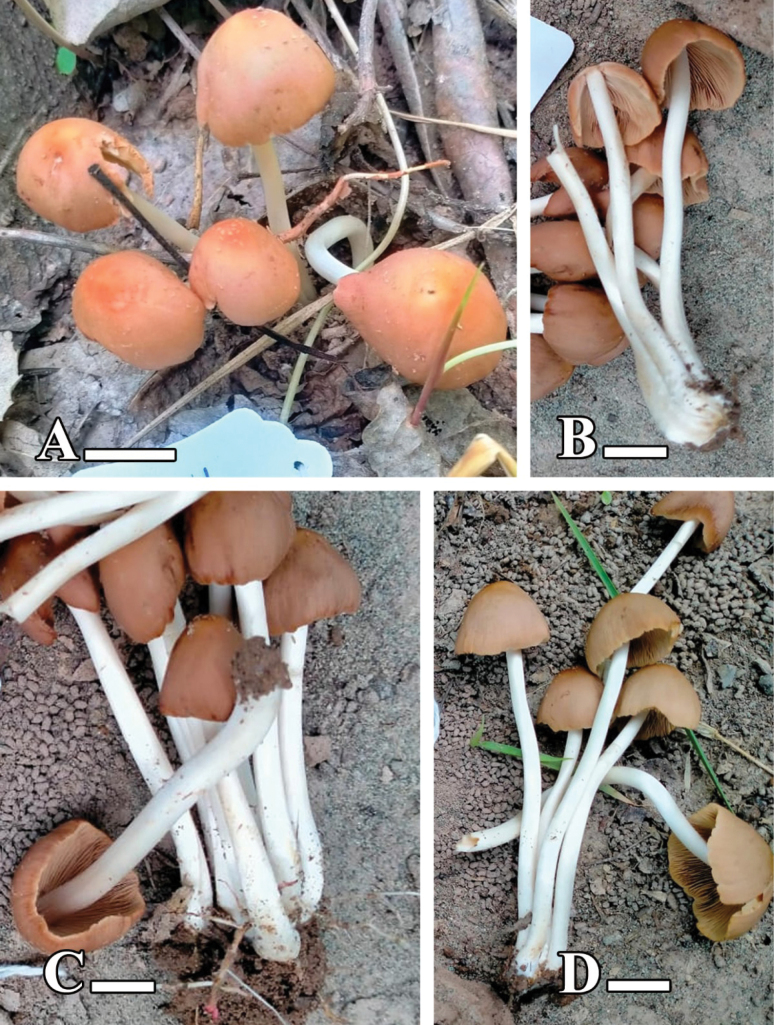
**A–D.** Basidiomata of *Candolleomyces kanhattiensis* sp. nov. (holotype LAH38586). Scale bars: 10 mm.

##### Description.

***Pileus*** 15–25 mm in diam., sub-globose to campanulate, convex, slightly umbonate, decurved with even to undulating margins, smooth surface, orange (5YR 7/6) to dull orange (7.5YR 7/4), with distinct dark orange (7.5YR 8/6) center. ***Lamellae*** adnate, narrow, even, with 3–7 tiers of lamellulae, dull orange (5YR 7/3), entire edge. ***Stipe*** 30–65 × 2–5 mm, cylindrical, central, equal, grayish white (N 8/0), shiny and smooth upwards, clavate base, narrow toward pileus. ***Annulus*** absent.

**Figure 8.**
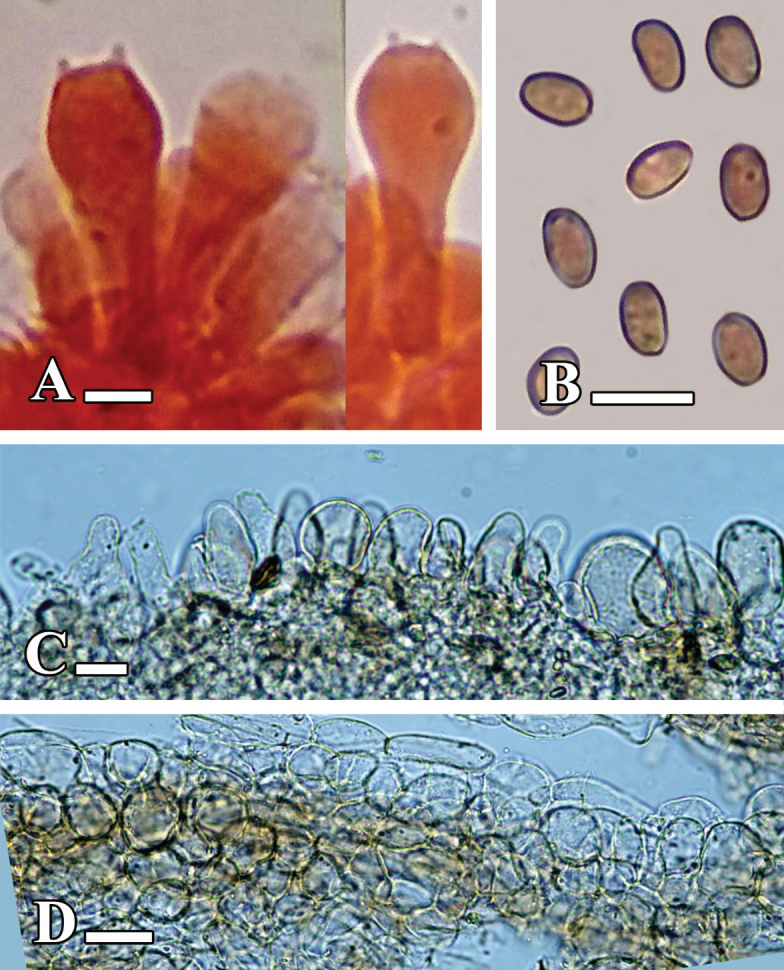
**A–D.** Microscopic structures of *Candolleomyces kanhattiensis* sp. nov. (holotype LAH38586). **A.** Basidia; **B.** Basidiospores; **C.** Cheilocystidia; **D.** Pileipellis. Scale bars: 10 µm (**A–D**).

***Basidiospores*** [150/3/2], (5.5–) 6–6.5 (–7) × (3.9–) 4–4.2 (–4.5) μm, Q = 1.541–1.55, Qav = 1.53, ellipsoid to ovoid, thick–walled, dull reddish in water, smooth, apiculate, germ-pore absent, inner contents visible. ***Basidia*** (18–) 19–22 (–24) × (6–) 7–8.5 (–9) μm, sub-clavate to clavate, cylindrical, hyaline in KOH, thin-walled, smooth, with 2–4 small sterigmata. ***Cheilocystidia*** (18–) 19–45 (–46) × (8.5–) 9–16 (–17) μm, avl × avw = 34 × 14 μm, ovoid, clavate, lageniform, broadly cylindrical, ellipsoid to broadly ellipsoid, utriform, globose to subglobose, thick–walled. ***Pleurocystidia*** absent. ***Pileipellis*** an irregular epithelium, 15–25 × 8–20 μm, made up of oblong, subglobose, ellipsoid to broadly ellipsoid, clavate hyaline to yellowish in water, thin-walled, smooth, up to 39 μm broad. ***Stipitipellis*** a cutis made up of thin-walled hyphae, 4–12 μm diam., smooth, hyaline in KOH, branched, clamp connections present.

##### Ecology and habitat.

Gregarious, rich clayey soils in damp places, some in rotting leaves, compost piles, or dead small sticks of wood.

##### Additional specimens examined.

Pakistan • Punjab Province, District Khushab, in groups on muddy nutrient-rich soil under *Dalbergia sissoo*, sometimes on woody debris, 05 Aug. 2023, Ayesha Bibi, KHU–33 (LAH38587). GenBank: PV265178 [ITS], PV265188 [LSU], PV357398 [*tef*-1α].

##### Notes.

Phylogenetically and morpho-anatomically, *Candolleomyces kanhattiensis* is closely related to *C. rubrobrunneus*, *C. denticulatus* nov. sp., and *C. pabbiensis*. It can be distinguished from *C. rubrobrunneus* by its sub-globose to campanulate, dull orange with dark orange center, decurved, undulating margin of pileus, shiny stipe with clavate base, greenish gray to light brownish gray basidiospores, and cylindrical, ellipsoid, and globose cheilocystidia. In contrast, *C. rubrobrunneus* has a conical, reddish brown to dark brown pileus with straight margins and a fibrillose to floccose surface, a pruinose apex of the stipe, broadly fusiform, capitate, flexuose cheilocystidia, and brownish yellow basidiospores (Nayana and Pradeep 2024). *Candolleomyces pabbiensis* exhibits a conical to broadly parabolic pileus with straight margins, grayish white lamellae, light brown basidiospores, and plump cheilocystidia with an obtuse to subobtuse apex (Haqnawaz et al. 2024b). *Candolleomyces denticulatus* has toothed margins, abundant squamules when young, a grayish white pileus, and reddish gray to yellowish gray basidiospores. Furthermore, the cheilocystidia are conical, and polymorphic caulocystidia are also present (in this study).

#### ﻿ Candolleomyces swaticus

Taxon classificationFungiAgaricalesPsathyrellaceae

Naseer, Haqnawaz & Khalid sp. nov.

DDB3C8BB-B6D9-5AF7-9F5B-B4EBF9CB4AD9

858287

Figs 9, 10

##### Etymology.

The species name “*swaticus*” (Latin) refers to the type locality, Swat.

##### Holotype.

Pakistan • Khyber Pakhtunkhwa Province: Swat (35°04'37"N, 72°24'33"E, 1480 m a.s.l.), on loamy soil, 11 Aug. 2016, Arooj Naseer, A. N. Khalid, SK–23 (LAH37132). GenBank: PV265182 [ITS], PV265191 [LSU].

##### Diagnosis.

*Candolleomyces swaticus* is different from *Candolleomyces candolleanus* by its large (20–60 mm), light gray with yellow-orange center, umbonate pileus, dull reddish-brown lamellae with 3–7 tiers, scales on stipe surface, bright brown basidiospores, capitate, lageniform, flexuose, tibiform, moniliform cheilocystidia.

##### Description.

***Pileus*** 20–60 mm diam., campanulate at young stage, convex to plane when old, broadly umbonate, margin straight, split and irregularly wavy, presence of veil elements, smooth dull surface; light gray (10 YR 8/1) with dull yellow orange (10 YR 7/3) center. ***Lamellae*** adnexed, narrow to broad, average, with 3–7 tiers of lamellulae, dull reddish brown (2.5 YR 5/4). ***Stipe*** 25–55 × 4–8 mm, central, flexuous, tomentose, equal in width, scaly, grayish white (N 8/0). ***Annulus*** absent.

**Figure 9.**
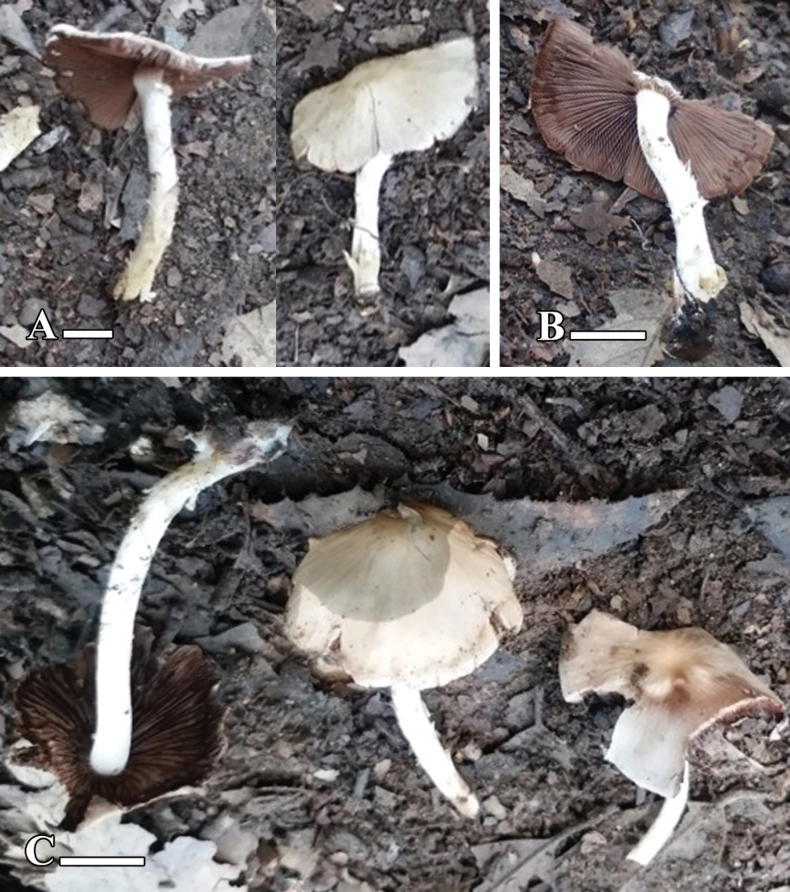
**A–C.** Basidiomata of *Candolleomyces swaticus* sp. nov. (holotype LAH37132). Scale bars: 20 mm.

***Basidiospores*** [150/3/3], (6–)6.5–7.5(–8) × (4–)4.2–4.8(–5) µm, Q = 1.3–1.8, Qav = 1.65, amygdaliform in side view, ovoid in frontal view, thick–walled, smooth, guttulate, central germ pore present, bright brown in water. ***Basidia*** 15–20 × 6.3–10.2 µm, clavate to broadly clavate, hyaline in water, thick–walled, smooth, with 3–4 sterigmata. ***Cheilocystidia*** (30–)31–49(–50) × (10–)11–19(–20) µm, utriform to slightly lecythiform, capitate, cylindrical to cylindrical with median constriction at upper side, narrowly lageniform to broadly lageniform, flexuose, broadly fusiform, tibiform, moniliform, hyaline to slightly yellowish in water, thick–walled. ***Pleurocystidia*** absent. ***Pileipellis*** an irregular epithelium, one layer, of thick–walled, 25–75 × 18–70 µm, globose to subglobose and ellipsoid to broadly ellipsoid, clavate to broadly clavate, cylindrical cells, hyaline to olive yellow in water. ***Stipitipellis*** a cutis, made of hyphae subregular, branched, 6–17 µm in diameter, thin-walled, septate, and hyaline to yellow in water. ***Caulocystidia*** not recorded. Clamp connections present in all tissues.

**Figure 10.**
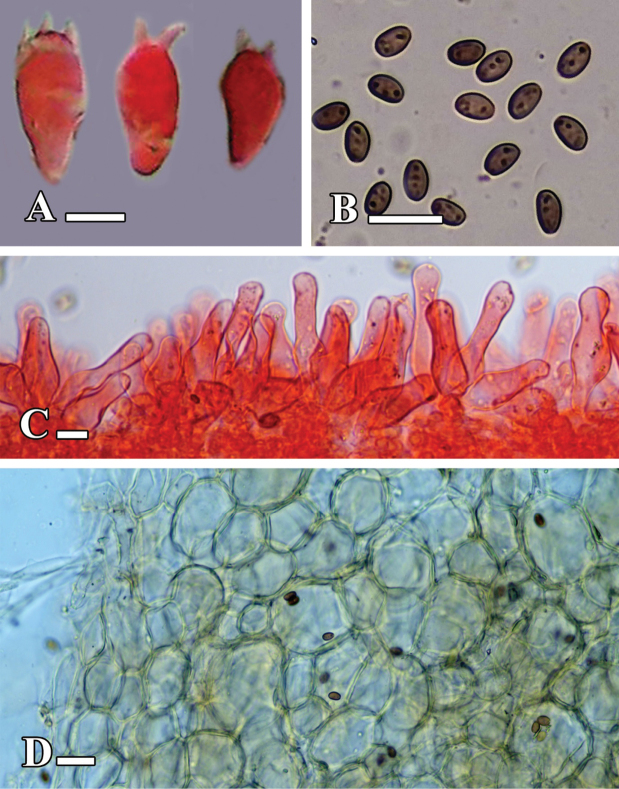
**A–D.** Microscopic structures of *Candolleomyces swaticus* sp. nov. (holotype LAH37132). **A.** Basidia; **B.** Basidiospores; **C.** Cheilocystidia; **D.** Pileipellis. Scale bars: 10 µm (**A–C**); 20 µm (**D**).

##### Ecology and habitat.

Terrestrial, gregarious, under a coniferous tree, on alluvium soil, during the rainy season.

##### Additional material examined.

Pakistan • Khyber Pakhtunkhwa Province: Swat (35°04'37"N, 72°24'33"E, 1480 m a.s.l.), on loamy soil, 15 Aug. 2019, Arooj Naseer, A. N. Khalid, SK–01 (LAH37131). GenBank: PV265183 [ITS], PV265190 [LSU].

##### Notes.

According to phylogenetic analyses, *Candolleomyces swaticus* is closely related to *C. candolleanus* and *C. badhyzensis*. Morpho-anatomically, the former is different from the latter by having a campanulate pileus when young, being light gray with a dull yellow-orange center, having absence of hairy material (vs. *C. candolleanus*, which is covered with hairy material), having the presence of a broad umbo, and having irregular, wavy cap margins. *Candolleomyces candolleanus* has a parabolic pileus at a young stage, a bright brown pileus, and straight to even cap margins (Wächter and Melzer 2020). The next closely related taxon is *Candolleomyces badhyzensis*, which differs from the new species by a broadly bell-shaped, yellowish-brown pileus and cylindrical to ellipsoid to phaseoliform, pale brown basidiospores (vs. amygdaliform to ovoid, grayish red) (Kalamees 1981).

### ﻿Key to the *Candolleomyces* species from Pakistan

**Table:** 

1	Annulus present	**2**
–	Annulus absent	**7**
2	Pileus width ≥ 50 mm	**3**
–	Pileus width ≤ 50 mm, with uplifted margins, light gray with dull orange center, long stipe (50–130 × 4–8 mm), capitate to lecythiform cheilocystidia	***C. conicus***
3	Umbo present	**4**
–	Umbo absent	**6**
4	Pileus not areolate	**5**
–	Pileus areolate, bilayered, decurved, and scalloped to cracked cap margins; serrate lamellae, capitate, lageniform, ovoid pedunculate cheilocystidia	***C. sindhudeltae***
5	Paraboloid to campanulate, grayish brown pileus, with appressed pale orange squamulose, caespitose base of stipe, absence of caulocystidia	***C. pallidosquamulosus***
–	White to dirty-white pileus, striations on surface, forked lamellae near margins, presence of caulocystidia	***C. sultanii***
6	Convex pileus, white to grayish with orange-yellow center, crenate to decurved margins of pileus	***C. pakistanensis***
–	Parabolic to campanulate, light purplish gray pileus with light brownish gray center, eroded lamellae, presence of caulocystidia	***C. undulatus***
7	Not Convex pileus	**8**
–	Convex pileus	**14**
8	Not Campanulate pileus	**9**
–	Campanulate pileus	**11**
9	White color of lamellae	**10**
–	Reddish gray lamellae, circular, applanate to plano-concave, centrally depressed, at center dull reddish brown pileus	***C. granulosis***
10	Hemispleuric pileus, covered with saccharine material	***C. efflorescens***
–	Conical to broadly parabolic pileus, covered with light gray squamules	***C. pabbiensis***
11	Even or regular cap margins	**12**
–	Striate margins, dull yellow-orange pileus, presence of caulocystidia	***C. campanulatus***
12	Light brownish gray pileus	**13**
–	Bright brown pileus covered with hairy material,	***C. candolleanus***
13	Conical pileus with light gray squamules, amygdaliform to phaseoliform basidiospores	***C. amygdaliformis***
–	Virgate pileus, umbonate, grayish red lamellae	***C. virgatus***
14	Umbo present	**15**
–	Umbo absent	**19**
15	Undulating or irregular cap margin	**16**
–	Crenate or striate cap margins	**18**
–	Glandular dotted surface of stipe with broad base, sphero-pedunculate cheilocystidia	***C. umbonatus***
16	Absence of fibrils on pileus and shiny stipe	**17**
–	Presence fibrils on pileus, stipe with bulbous base	***C. asiaticus***
17	Orange to dull orange, with distinct dark orange center, dull orange lamellae, ovoid, ellipsoid, globose cheilocystidia	***C. kanhattiensis***
–	Light gray with dull yellow orange center, dull reddish brown lamellae, lecythiform, capitate, broadly fusiform, tibiform, moniliform cheilocystidia	***C. swaticus***
18	Glandular dotted surface of stipe with broad base, dark reddish to brown lamellae with yellow orange edges, spheropedunculate cheilocystidia, dark brown basidiospores	***C. umbonatus***
–	Shiny surface and long (40–65 mm) stipe, light yellowish basidiospores	***C. iqbalii***
19	Straight margin	**20**
–	Decurved margins, light brown to golden brownish pileus, long (55–75 mm) stipe	***C. parvipileus***
20	Adnexed or adnate lamellae	**21**
–	Emarginate lamellae, striations toward the margin of pileus, obovoid pedunculate cheilocystidia	***C. kotadduensis***
21	Crenate or striate margin	**22**
–	Split in symmetry to toothed margin, forked, grayish brown lamellae with grayish white edge	***C. denticulatus***
22	Fimbriate, 1–5 tiers of lamellulae, marginate base of stipe, caulocystidia present	***C. crenatus***
–	1–2 tiers of lamellulae clavate base of stipe, absence of caulocystidia	***C. albogranulosus***

## ﻿Discussion

According to phylogenetic analyses and morpho-anatomical characterization, all taxa—*Candolleomyces conicus*, *C. denticulatus*, *C. kanhattiensis*, and *C. swaticus*—which are new to science, formed separate branches from their closest species with significant differences. In comparison to each other, all new taxa show unique characters: *C. conicus* has large basidiomata, a conical cap with an uplifted margin, an areolate pileus, lecythiform to lageniform cheilocystidia, and growth around the dead trunk of *Dalbergia sissoo*; *C. denticulatus* has toothed cap margins, grayish white edges of lamellae, and growth under *Punica granatum*; *C. kanhattiensis* has a subglobose pileus with decurved margins and a shiny stipe with clavate base; and *C. swaticus* has a distinct broad umbo, a light gray pileus with a yellow-orange center, dull reddish-brown lamellae, and occurrence in mountainous areas under coniferous trees. *Candolleomyces conicus*, *C. denticulatus*, and *C. swaticus* are terrestrial, while *C. kanhattiensis* is lignicolous. *Candolleomyces conicus*, *C. denticulatus*, and *C. kanhattiensis* are reported from subtropical regions, while *C. swaticus* is reported from tropical regions.

In a previous study, certain species of *Candolleomyces* exhibited significant differences in their ITS regions. Nevertheless, some *Candolleomyces* species with high similarity (more than 99%) are still treated as separate species based on distinct morpho-anatomical characters (Bau and Yan 2021). Therefore, some species are identified based only on the ITS region, while others require additional regions (LSU, *tef*-1α, and β-tub) for identification. The addition of four new species to the fungal flora of Pakistan makes a significant contribution, increasing the total number of species in the country to 23, which highlights the rich diversity of the genus in the region. This discovery not only expands our understanding of the *Candolleomyces* genus but also emphasizes the vast diversity of fungal species that remain to be explored. It also serves as evidence of mycologists’ ongoing efforts to uncover the hidden diversity of fungal species and advance our knowledge of the natural world. Consequently, further extensive systematic surveys are imperative to explore, understand, and document the full extent of *Candolleomyces* diversity and distribution within the region.

## Supplementary Material

XML Treatment for Candolleomyces conicus

XML Treatment for Candolleomyces denticulatus

XML Treatment for Candolleomyces kanhattiensis

XML Treatment for Candolleomyces swaticus

## ﻿Additional information

### Use of AI

No use of AI was reported.

### Author ORCIDs

Muhammad Haqnawaz https://orcid.org/0000-0003-4667-242X

Qirui Li https://orcid.org/0000-0001-8735-2890

Aysha Bibi https://orcid.org/0000-0002-0318-3151

Arooj Naseer https://orcid.org/0000-0002-4458-9043

Abdul Nasir Khalid https://orcid.org/0000-0002-5635-8031

### Data availability

All of the data that support the findings of this study are available in the main text.
